# Characterization of human breast cancer epithelial cells (HBCEC) derived from long term cultured biopsies

**DOI:** 10.1186/1756-9966-28-127

**Published:** 2009-09-14

**Authors:** Ralf Hass, Catharina Bertram

**Affiliations:** 1 Clinic of Obstetrics and Gynecology, Biochemistry and Tumor Biology Lab, Medical University, Hannover, Germany

## Abstract

**Introduction:**

For a more individualized therapeutic approach we explored a protease-free method to culture primary cells from breast cancer biopsies.

**Methods and Results:**

Tumor tissue from breast cancer patients after surgery was cultured *ex vivo *without enzymatic digestion for more than one year and revealed the continuous outgrowth of adherent and proliferating primary cell populations. Immunofluorescence staining of these human breast cancer-derived epithelial cells (HBCEC) and quantification by flow cytometry revealed nearly exclusively cytokeratin-expressing cells. Analysis of surface markers during long term tumor culture of primary HBCEC (more than 476d) demonstrated a prominent expression of CD24, CD44 and MUC1 (CD227). According to aging markers, expression of senescence-associated β-galactosidase revealed little if any positive staining in a primary tumor-derived HBCEC population after 722d in culture, whereas the majority of normal human mammary epithelial cells (HMEC) demonstrated senescent cells already after a culture period of 32d. In this context, HBCEC populations derived from a tumor culture after 152d and 308d, respectively, exhibited a significant telomerase activity, suggesting continuous proliferative capacity. Treatment with several chemotherapeutic compounds and their combinations revealed distinct cytotoxic effects among HBCEC from different breast cancer patients, indicating an individualized response of these tumor-derived primary cells.

**Conclusion:**

The protease-free outgrowth of primary HBCEC offers a patient-specific approach to optimize an individually-designed cancer therapy. Moreover, HBCEC from long term breast tumor tissue cultures resemble tumor cell-like properties by an intact ECM formation and a stable cell surface protein expression providing a reproducible screening platform to identify new biomarkers and to test new therapeutics in individual tumor samples.

## Background

Individual primary cultures of tissue biopsies from breast cancer patients represent an alternative model for *in vitro *studies as compared to the use of immortalized breast cancer cell lines. Thus, immortalization and genetic drifts in cell lines have to be extrapolated for the appropriate clinical application [1]. A variety of previous investigations, using enzymatic digestion of the appropriate breast tissue, extracted normal as well as malignant breast epithelial cells and reported distinct properties of these isolated primary cells [1-6]. It has been indicated that the culture of isolated cells from protease-digested solid tumors includes the risk of an overgrowth by fibroblasts or stromal cells [1,7], demanding subsequent selective culture conditions. Growth of primary breast epithelial cells, also termed as human mammary epithelial cells (HMEC) [3,4], and breast cancer-derived epithelial cells (HBCEC) is preferentially stimulated in serum-free medium conditions and thus allows selection among fibroblasts [8,9]. The enzymatic and mechanical approach to isolate mammary cells from tissues also revealed certain mammary stem/progenitor cells in suspension culture [10,11]. These mammary stem/progenitor cells can appear in multicellular aggregates termed as mammospheres with proliferative capacity for self-renewal and the potential to generate differentiated progeny [12]. Thus, distinct culture conditions of mammospheres provide the ability to induce differentiation into ductal, myoepithelial, and alveolar mammary cells, respectively [13]. A variety of markers, including morphology, growth properties [3-5], specific antigen and cytokeratin expression [1,7] as well as metabolic alterations during aging [2] have been characterized in HMEC and in initially cultured breast tumor cells. For a more general detection and characterization of malignant tumor cells in solid human tumors, a cytopathological examination and the measurement of telomerase activity was suggested [14].

Enzymatic digestion of breast tumor tissue by distinct proteases to obtain single cells and further subculture by trypsinization include non-specific proteolytic effects which may interfere with intracellular signaling mechanisms and cell cycle progression [15,16]. Recent studies have demonstrated that the architecture of the mammary tissue requires cell adhesion proteins, in particular E- and P-cadherins, which play an important role to maintain normal mammary cell functions and proliferation [17]. Moreover, transmembrane adhesion molecules such as integrins and their interaction with the cytoskeleton are essential for normal as well as breast cancer cells, respectively [15,18], and the epithelial cells are highly susceptible to alterations of the extracellular matrix (ECM) [10,16]. This suggests, however, that enzymatic degradation of parts of this sensitive ECM network may abolish distinct signaling pathways or induce a certain aberrant signal transfer in breast tumor tissue. Indeed, previous work has demonstrated that the detection and function of certain marker proteins in HBCEC was restricted to short term cultures and characteristics of the original tumor tissue could not be preserved during extended cultivation [7].

In the present study we characterize primary human breast cancer epithelial cells (HBCEC), derived from a direct tumor tissue outgrowth without any protease digestion. These primary HBCEC cultures could serve as a patient-specific approach to optimize an individually-designed cancer therapy. Moreover, the tumor tissues can be maintained for long term in culture and the obtained HBCEC cultures represent typical tumor cell properties in contrast to limited cell divisions of normal HMEC, thus providing a potential testing platform to investigate new therapeutic strategies.

## Materials and methods

### Individual mammary tumor-derived cell cultures

Small tissue pieces from 8 different breast cancer patients were collected during surgery and pathologically characterized as ductal carcinomas, respectively. Informed written consent was obtained from each patient for the use of individual biopsy material and the study has been approved by the Institutional Review Board, Project #3916 on June 15th, 2005. The tissue samples were cut into small blocks of approximately 1 mm^3 ^and washed extensively in PBS to remove blood cells and cell debris. After negative testing for HIV-1, hepatitis B & C, bacteria, yeast and fungi, respectively, the tissue pieces of the mammary tumors were incubated using plain uncoated plastic dishes (Nunc GmbH, Langenselbold, Germany) in serum-free mammary epithelial cell growth medium (MEBM) (PromoCell GmbH, Heidelberg, Germany), supplemented with 52 μg/ml of bovine pituary extract, 0.5 μg/ml of hydrocortisone, 10 ng/ml of human recombinant epidermal growth factor and 5 μg/ml of human recombinant insulin in a humidified atmosphere at 37°C. Half of the cell culture medium was replaced about every fourth day and the other half was used as conditioned medium. Under these conditions, an outgrowth of primary tumor-derived cells was observed, which were adherent to the tumor tissue blocks and to each other. In the subconfluent growth phase the tumor tissue pieces were separated from the culture and placed into a separate culture dish to allow further outgrowth of primary tumor cells. The remaining tumor-derived cells were used for the appropriate assays.

### Normal human mammary epithelial cell cultures

Primary cultures of normal human mammary epithelial cells (HMEC) were isolated from a 50 year old caucasian female and commercially provided by BioWhittaker Inc. (Walkersviell, MD, USA) as culture passage 7 (Lot #1F1012). HMEC were tested positive for cytokeratins 14 and 18 and negative for cytokeratin 19, respectively. They were performance tested and tested negative for HIV-1, hepatitis B & C, mycoplasma, bacteria, yeast and fungi. HMEC were seeded at 4,500 cells/cm^2^, cultured in MEBM (PromoCell) and the appropriate medium of each culture was replaced every two to three days. At subconfluent conditions the cells were subcultured by incubation with 0.025%/0.01% trypsin/EDTA (PromoCell) for about 6 min/37°C until the cells detached. Thereafter, immediate addition of trypsin neutralization solution (TNS) from soybean was required to inactivate the trypsin followed by subsequent centrifugation (220 g/6 min). The pelleted cells were resuspended in new medium at about 4,500 cells/cm^2 ^and cultured further on in the next passage number. Subcultured cells required about 24 h to recover and resume growth.

### MCF-7 cell line

Human MCF-7 mammary gland adenocarcinoma cells originally isolated from a 69 year old caucasian woman with several characteristics of differentiated mammary epithelium were derived from the American Type Culture Collection (ATCC #HTB-22) as passage 146 or earlier and cultured inititally at about 1,500 cells/cm^2 ^in DMEM-medium (Invitrogen GmbH, Karlsruhe), including 10% (v/v) heat-inactivated fetal calf serum (FCS) (Biochrom KG), 2 mM L-Glutamin (Invitrogen), 1 mM Na-Pyruvat (Invitrogen) and 1 mM Penicillin/Streptomycin (Invitrogen).

### MDA-MB-231 cell line

Human MDA-MB-231 mammary gland adenocarcinoma cells isolated as one of a series of breast tumor lines from pleural effusions of a 47 year old caucasian female were derived from the ATCC (#HTB-26) and cultivated inititally at about 1,500 cells/cm^2 ^in Leibovitz's L-15-medium (Invitrogen) with 10% (v/v) FCS, 2 mM L-Glutamin and 1 mM Penicillin/Streptomycin.

### Electron microscopy

The mammary tumor tissues were cultured on appropriate microscope slides for scanning (SEM) and transmission electron microscopy (TEM), respectively. Following *ex vivo *outgrowth of tumor-derived cells, the individual cultures were fixed on these slides in a solution containing 3% glutaraldehyde in 0.1 M sodium cacodylate, pH 7.4 for at least 24 h. Thereafter, the samples were postfixed in 1% OsO_4 _in H_2_O before being dehydrated in an ethanol gradient. For SEM, critical point-dried specimen were coated with gold-palladium (SEM coating system E5400, Polaron, Watford, UK) and examined in a JEOL SSM-35CF scanning electron microscope at 15 kV.

For TEM, the ethanol dried mammary tumor tissues were embedded in Epon. Ultrathin sections were stained with uranyl acetate and lead acetate and examined in a Philips CM10 electron microscope, operated at 80 kV.

### Immunofluorescence

Mammary tumor-derived cells were cultured onto microscope slides, washed 3× with PBS/Tween-20 for 5 min, and air-dried for 60 min. Thereafter, the samples were fixed with ice-cold acetone for 10 min and rehydrated in PBS for 5 min. After treatment with PBS/5% (w/v) BSA for 10 min to block non-specific binding-sites, the samples were incubated with a mouse anti-vimentin antibody (cloneV9 (1:100); Dako, Hamburg, Germany) for 30 min. Following three washes with PBS/Tween-20 for 5 min, respectively, the samples were incubated with a TRITC-labelled anti-mouse secondary antibody ((1:40); Dako) for 90 min. Another 3 washes with PBS/Tween-20 were performed for 5 min, and after blocking with a mouse serum ((1:40); Dako), the samples were incubated with a FITC-conjugated monoclonal anti-pan cytokeratin antibody (clone MNF116 (1:20); Dako) for 90 min. After further three washes with PBS/Tween-20 for 5 min, the samples were incubated with a DAPI-containing medium (Dako), which simultaneously preserve the samples for subsequent immunofluorescence microscopy. For background and control staining, the tumor-derived cell passages were incubated with mouse sera of the appropriate IgG subclass instead of using the primary antibodies. Fluorescence microscopy was performed with an Olympus SIS F-View II CCD-camera associated with an Olympus IX-50 fluorescence microscope (Olympus, Hamburg, Germany). The fluorescence image analysis and the fluorescence overlay image was obtained with the SIS bundle analySIS'B image software (Olympus). Accordingly, cytokeratin filaments demonstrated green, vimentin filaments red, and DNA within the cell nuclei blue fluorescence, respectively.

### Cytokeratin and vimentin quantification by flow cytometry

About 5 × 10^5 ^mammary tumor-derived cells were fixed by consecutive addition of ice-cold ethanol to a final concentration of 70% (v/v). Thereafter, the cells were stored at 4°C for at least 24 h. Following 2× washes with PBS, the cells were incubated with a monoclonal anti-pan-cytokeratin (clone MNF116; Dako), anti-vimentin antibody (clone V9; Dako) and anti-desmin antibody (clone D33; DakoCytomation), respectively, for 30 min at 4°C. After washing with PBS the samples were incubated with a RPE-conjugated F(ab')2 fragment of goat anti-mouse immunoglobulin (1:10 (v/v); Dako) for 30 min at 4°C in the dark. Incubation of the cells with the secondary antibodies alone was used as a negative control and background staining. Following three washes with PBS the samples were analyzed in a Galaxy FACScan (Dako) using FloMax analysis software (Partec GmbH, Münster).

### Flow cytometry analysis of surface marker expression

Tumor-derived HBCEC obtained from the same tumor piece after tissue culture for 176d and for 462d, respectively, were trypsinized and fixed in 70% ice-cold ethanol at 4°C for 24 h. Thereafter, the cells were washed twice with PBS and incubated with the FITC-conjugated CD24, CD44, and CD227 antibodies (all from BD Biosciences, Heidelberg, Germany, according to the manufacturer's protocol) and the isotype-specific negative controls (Dako), for 30 min at room temperature. After two additional washing steps, the cells were measured with a Galaxy FACScan (Dako) using FloMax analysis software (Partec).

### SA-β-galactosidase assay

The mammary tumor-derived cells after 722d of tumor tissue culture were compared to normal HMEC in passage 16 after 32d. The cells were fixed and stained against the senescence-associated β-galactosidase (SA-β-gal) for 24 h/37°C in the dark according to the manufacturers protocol and recommendations (Cell Signaling Technology, Danvers, MA, USA). Following two washes with PBS the differentially-stained cell cultures were documented by phase contrast microscopy with an Olympus IX50 microscope using the Olympus imaging software cell^B ^(Olympus).

### Telomerase (TRAP-)assay

The TRAPEZE^® ^Gel-Based Telomerase Detection assay (Chemicon International, Temecula, CA, USA) was performed according to the manufacturer's protocol using the isotopic detection. HBCEC populations from two different patients were tested, whereby one was obtained after 308d of tumor tissue culture. HBCEC from the other patient were collected after 152d of tumor tissue culture both, by trysinization or by scraping with a rubber policeman. The human embryonic kidney (HEK) cell line 293T was obtained by trypsinization of a steady state culture and used as a positive control. Briefly, HBCEC and 293T control cells were washed with ice-cold PBS and homogenized in 100 μl ice-cold 1× CHAPS lysis buffer (Chemicon). After incubation for 30 min on ice, the homogenates were centrifuged (12000 g/30 min/4°C) and the supernatants were transferred to a new tube and subjected to a protein quantification measurement using the BCA protein assay. According to the Chemicon protocol, the TS primer were radioactively end-labeled with γ-^32^P-ATP before the telomeric repeat amplification reaction was set up to allow the isotopic detection (see Chemicon protocol). Each assay included an internal standard (36 bp band) to control the amplification efficiency. A primer-dimer and PCR contamination control was performed by substituting the cell extract with 1× CHAPS lysis buffer. For data analysis, 25 μl of the amplified product were loaded on a 12.5% non-denaturating PAGE in 0.5× TBE buffer and eventually visualized using a PhosphorImager (GE Healthcare, Freiburg, Germany).

### ATP release assay following treatment with chemotherapeutic compounds

The effects of chemotherapeutic reagents on two different primary HBCEC were analyzed using the luciferin-luciferase-based ATP tumor chemosensitivity assay (ATP-TCA). Cytotoxicity was determined by measuring the luminescence of luciferin that is proportional to the ATP-release of intact cells. Triplicates of about 1.5 × 10^4 ^HBCEC were incubated with different concentrations of chemotherapeutic compounds (Taxol (Bristol-Myers-Squibb); Epothilone A and B (kind gift from Prof. G. Höfle, Helmholtz Center for Infection Research, Braunschweig, Germany); Epirubicin (Pharmacia&Upjohn); Doxorubicin (Sigma)) in a 96-well plate for 6d at 37°C, 5% CO_2_. The ATP-TCA assay was performed according to the manufacturer's protocol (DCS Diagnostica GmbH, Hamburg, Germany) using non-treated cells and cells incubated with the Maximum ATP-inhibitor Solution (DCS) as controls together with an ATP standard. Following lysis of the tumor cells with an extraction buffer (DCS), the luminescence was measured in a fluoro/luminometer (Fluoroskan Ascent FL Labsystems, Thermo Scientific, Dreieich, Germany) after addition of the luciferin-luciferase reagent and the percentage of intact (viable) cells was calculated using the Ascent software (Thermo Scientific).

## Results

**Figure 1:**
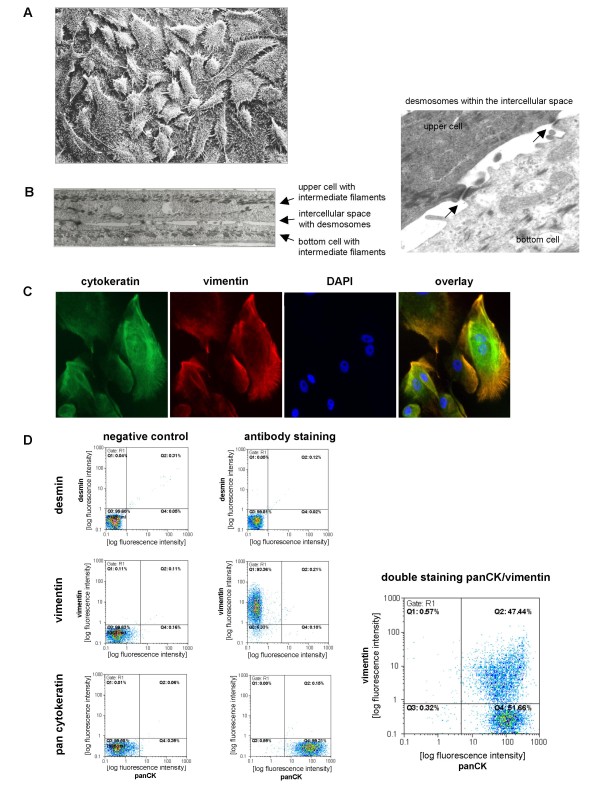
**Characterization of primary human breast cancer epithelial cells (HBCEC)**. **A**. Scanning electron micrographs of human breast cancer-derived cell cultures. The cells are squamous with many short and thin processes and grow upon each other. **B**. Ultrathin sections of two human breast cancer-derived cells, which partially overlap and are connected by desmosomes. The cells contain bundles of intermediate filaments and cytoplasmic vacuoles, whereas organelles are almost absent. In the right transmission micrograph, two squamous cell processes are connected by desmosomes and bundles of intermediate filaments are orientated in parallel to the cell surface. **C**. Immunofluorescence of intermediate filaments. Nuclei became visual using DAPI and the intermediate filament proteins cytokeratin (green) and vimentin (red) were detected by FITC-conjugated mouse anti-cytokeratin and mouse anti-vimentin antibody, respectively. **D**. Quantification of cytokeratin, vimentin and desmin expression by flow cytometric analysis. About 99% of the HBCEC population stained positive for cytokeratin, whereof some were positive for both, cytokeratin and vimentin intermediate filament proteins. Expression of desmin intermediate filaments remained undetectable. The FITC-labeled IgG control and the secondary antibody control served as background staining balance.

The *ex vivo *culture of tumor tissue from breast cancer patients after surgery was associated with the outgrowth of adherent human breast cancer epithelial-like cells (HBCEC) and demonstrated a massive extension of cytoplasmic protrusions similar to the morphology as described for normal human mammary epithelial cells (HMEC) (Fig. 1A) [2]. In contrast to the HMEC growth as a monolayer, HBCEC cultures revealed a multilayer cell growth and were connected to each other by numerous desmosomes (Fig. 1B).

Immunofluorescence staining exhibited a significantly green-colored cytokeratin expression within all of the HBCEC cultures (Fig. 1C), demonstrating epithelial-like cells rather than a contamination with other cell types such as fibroblasts. Additional testing for the fibroblast-specific prolyl-4-hydroxylase remained below detection limit in HBCEC cultures (data not shown). Co-immunofluorescence analysis was performed with red-labeled vimentin, which also appeared in certain cells (Fig. 1C). Blue DAPI staining of the nuclei and an overlay image revealed a co-expression of cytokeratin and vimentin in a variety of cells, demonstrating a different intracellular localization of these intermediate filaments (Fig. 1C). Quantification of vimentin and cytokeratin expression by flow cytometry revealed about 99% of cytokeratin-positive cells, whereby about 32% of this population demonstrated both, vimentin-positive and cytokeratin-positive cells, respectively (Fig. 1D). In contrast, flow cytometry analysis of desmin filaments which are predominantly observed in myoepithelial and myofibroblastic cell phenotypes revealed no detectable staining of either culture (Fig. 1D). Although the amount of vimentin may vary throughout different HBCEC cultures, cytokeratin levels were always detected at 95% or higher. Moreover, while the expression of intermediate filaments (Fig. 1C and 1D) was obtained from primary tumor cells after 34d, longer term culture remained stable displaying a similar pattern of intermediate filaments (data not shown). Together, these data suggested an almost exclusively epithelial-like cell population of HBCEC.

**Figure 2:**
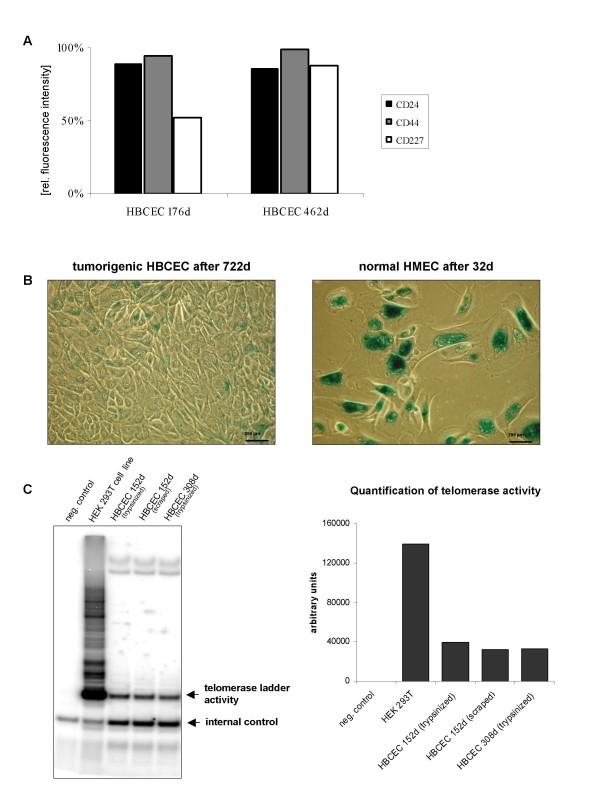
**Surface marker expression, SA-β-gal staining and telomerase activity in HBCEC**. **A**. Determination of the percentage of cell surface marker expression in HBCEC at different ages. Expression of the surface marker proteins CD24, CD44, CD227 was maintained during long term culture of HBCEC. Whereas CD24 and CD44 were similarly expressed after 176d and 462d, CD227 increased from 52% to 88% in HBCEC 462d. The flow cytometry measurements varied by about 8%. **B**. SA-β-gal staining of primary HBCEC and HMEC cultures. Staining for SA-β-gal of a HBCEC population after 722d in culture revealed little if any positive cell. Normal HMEC in passage 16, however, displayed already predominantly enlarged senescent cells after 32d, demonstrated by the dark-green stain (bar = 200 μm). **C**. Telomerase (TRAP-)assay of primary cultures from breast cancer biopsies. Telomerase activity was analyzed according to the Telomeric Repeat Amplification Protocol (TRAP). HBCEC populations demonstrated telomerase activity independent of the age of the culture and the harvest method. The human embryonic kidney (HEK) 293T cell line was used as a positive control and 1× CHAPS buffer served as a negative control. Quantification was performed using densitometric analysis.

To evaluate cell surface markers during long term culture of the breast tumors, an HBCEC population after 176 days was analyzed for CD24, CD44 and CD227, respectively, and compared to a tumor culture of the same patient after 462 days (Fig. 2A). Thus, CD24 was expressed in 89% of 176d HBCEC and in 86% of 462d HBCEC. Moreover, CD44 appearance was detectable in 94% of 176d HBCEC and in 99% of 462d HBCEC, suggesting little if any changes of both, CD24 and CD44 during long term tumor culture (Fig. 2A). In contrast, expression of the CD227 (MUC1) surface protein significantly increased from 52% in 176d HBCEC to 88% in 462d HBCEC (Fig. 2A).

Further characterization of the HBCEC cultures was performed to determine aging cells in a senescence-associated β-galatosidase (SA-β-gal) assay as compared to normal post-selection human mammary epithelia cells (HMEC) (Fig. 2B). Thus, SA-β-gal staining of primary cultures from breast cancer biopsies after 722d demonstrated majorly small young cells and only occasional positively-stained senescent cells in contrast to normal post-selection HMEC (P16) after 32d with almost exclusively large SA-β-gal positive senescent cells (Fig. 2B).

Evaluations by video microscopy (data not shown) and previous work have demonstrated the proliferative capacity of small-seized young breast epithelial cells [5]. Consequently, telomerase assays were performed and revealed telomerase activity of autonomously proliferating cells in all HBCEC populations (Fig. 2C). The human embryonic kidney (HEK) 293T cell line served as a positive control and the buffer was used as a negative control. Together, these findings suggested a sustained expression of epithelial stem cell-like markers in HBCEC paralleled by only occasional senescence and a marked telomerase activity.

**Figure 3:**
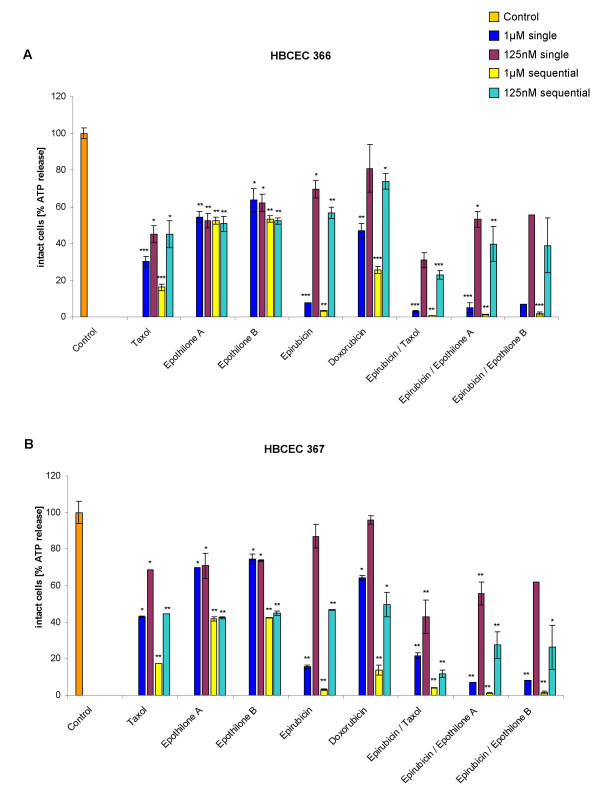
**Chemotherapeutic effects on HBCEC from breast cancer patients**. HBCEC derived from a 40 year-old (HBCEC 366) **(Fig. 3A) **and a 63 year-old (HBCEC 367) **(Fig. 3B) **woman both with ductal breast carcinoma, the breast cancer cell lines MCF-7 **(Fig. 4A) **and MDA-MB-231 **(Fig. 4B)**, and normal HMEC in passage 16 **(Fig. 5) **were incubated with a single dose of 1 μM (blue bars) and 125 nM (red bars) of appropriated chemotherapeutic compounds (Taxol, Epothilone A, Epothilone B, Epirubicin, Doxorubicin) and certain anthracyclin combinations (Epirubicin/Taxol, Epirubicin/Epothilone A, Epirubicin/Epothilone B) for 6d, respectively. Alternatively, the drugs were replaced after 3d, resulting in a similar 6d (= 2× 3d) incubation of the same compounds, using concentrations of 1 μM (yellow bars) and 125 nM (turquoise bars), respectively. Whereas the higher concentration of 1 μM was generally more effective, this was further promoted by a sequential treatment. Moreover, the HBCEC populations revealed distinct effects to the anticancer drugs Epothilone A and B, suggesting an individual responsiveness specific for the appropriate patient (Fig. 3A, B). Similarly, Epothilone A and B exhibited different effects on the two breast carcinoma cell lines. Furthermore, the non-metastatic MCF-7 cell line displayed an overall increased sensitivity to the administered drugs or drug combinations as compared to the highly metastatic MDA-MB-231 cells (Fig. 4A, B). HMEC (P16) demonstrated reduced cytotoxic effects of the chemotherapeutics as compared to the HBCEC cultures (Fig. 5). Data represent the mean +s.d. (n = up to 5 replicates). P values were calculated by the unpaired T-test according to the appropriate untreated control cells (Control). Results were considered as statistically significant when P value was < 0.5 (*P < 0.5; **P < 0.05; ***P < 0.005).

**Figure 4:**
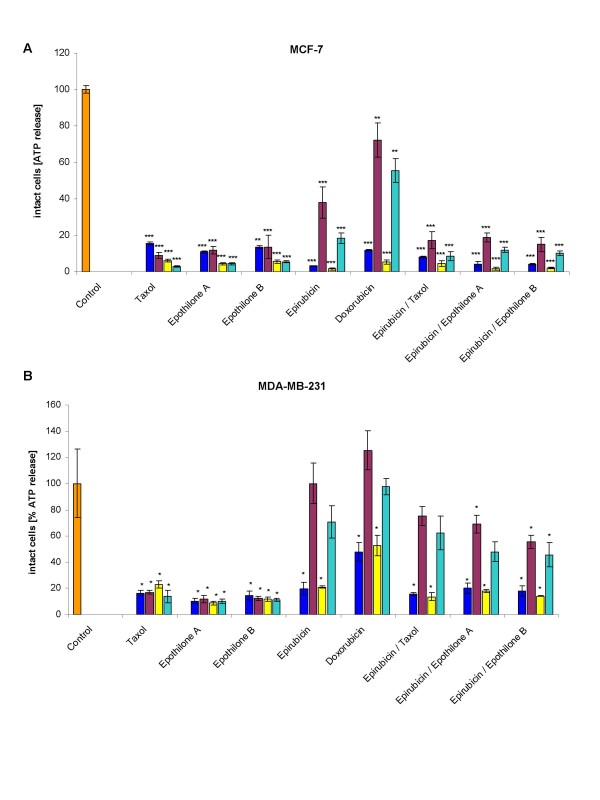
**Chemotherapeutic effects on HBCEC, breast cancer cell lines**. HBCEC derived from a 40 year-old (HBCEC 366) **(Fig. 3A) **and a 63 year-old (HBCEC 367) **(Fig. 3B) **woman both with ductal breast carcinoma, the breast cancer cell lines MCF-7 **(Fig. 4A) **and MDA-MB-231 **(Fig. 4B)**, and normal HMEC in passage 16 **(Fig. 5) **were incubated with a single dose of 1 μM (blue bars) and 125 nM (red bars) of appropriated chemotherapeutic compounds (Taxol, Epothilone A, Epothilone B, Epirubicin, Doxorubicin) and certain anthracyclin combinations (Epirubicin/Taxol, Epirubicin/Epothilone A, Epirubicin/Epothilone B) for 6d, respectively. Alternatively, the drugs were replaced after 3d, resulting in a similar 6d (= 2× 3d) incubation of the same compounds, using concentrations of 1 μM (yellow bars) and 125 nM (turquoise bars), respectively. Whereas the higher concentration of 1 μM was generally more effective, this was further promoted by a sequential treatment. Moreover, the HBCEC populations revealed distinct effects to the anticancer drugs Epothilone A and B, suggesting an individual responsiveness specific for the appropriate patient (Fig. 3A, B). Similarly, Epothilone A and B exhibited different effects on the two breast carcinoma cell lines. Furthermore, the non-metastatic MCF-7 cell line displayed an overall increased sensitivity to the administered drugs or drug combinations as compared to the highly metastatic MDA-MB-231 cells (Fig. 4A, B). HMEC (P16) demonstrated reduced cytotoxic effects of the chemotherapeutics as compared to the HBCEC cultures (Fig. 5). Data represent the mean +s.d. (n = up to 5 replicates). P values were calculated by the unpaired T-test according to the appropriate untreated control cells (Control). Results were considered as statistically significant when P value was < 0.5 (*P < 0.5; **P < 0.05; ***P < 0.005).

**Figure 5:**
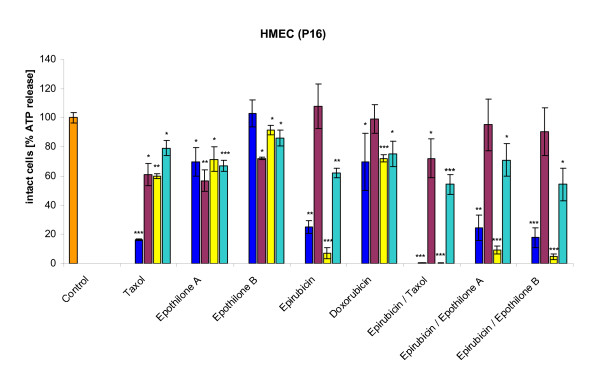
**Chemotherapeutic effects on normal human mammary epithelial cells in passage 16 (HMEC P16)**. HBCEC derived from a 40 year-old (HBCEC 366) **(Fig. 3A) **and a 63 year-old (HBCEC 367) **(Fig. 3B) **woman both with ductal breast carcinoma, the breast cancer cell lines MCF-7 **(Fig. 4A) **and MDA-MB-231 **(Fig. 4B)**, and normal HMEC in passage 16 **(Fig. 5) **were incubated with a single dose of 1 μM (blue bars) and 125 nM (red bars) of appropriated chemotherapeutic compounds (Taxol, Epothilone A, Epothilone B, Epirubicin, Doxorubicin) and certain anthracyclin combinations (Epirubicin/Taxol, Epirubicin/Epothilone A, Epirubicin/Epothilone B) for 6d, respectively. Alternatively, the drugs were replaced after 3d, resulting in a similar 6d (= 2× 3d) incubation of the same compounds, using concentrations of 1 μM (yellow bars) and 125 nM (turquoise bars), respectively. Whereas the higher concentration of 1 μM was generally more effective, this was further promoted by a sequential treatment. Moreover, the HBCEC populations revealed distinct effects to the anticancer drugs Epothilone A and B, suggesting an individual responsiveness specific for the appropriate patient (Fig. 3A, B). Similarly, Epothilone A and B exhibited different effects on the two breast carcinoma cell lines. Furthermore, the non-metastatic MCF-7 cell line displayed an overall increased sensitivity to the administered drugs or drug combinations as compared to the highly metastatic MDA-MB-231 cells (Fig. 4A, B). HMEC (P16) demonstrated reduced cytotoxic effects of the chemotherapeutics as compared to the HBCEC cultures (Fig. 5). Data represent the mean +s.d. (n = up to 5 replicates). P values were calculated by the unpaired T-test according to the appropriate untreated control cells (Control). Results were considered as statistically significant when P value was < 0.5 (*P < 0.5; **P < 0.05; ***P < 0.005).

Individually-derived HBCEC populations from cultured breast cancer biopsies were tested for their response to distinct chemotherapeutic compounds and combinations. Thus, HBCEC populations (39d) from tumor biopsies of a 40 year-old (Fig. 3A) and HBCEC populations (34d) a 63 year-old patient (Fig. 3B) were treated with 125 nM and 1 μM of Taxol, Epothilone A, Epothilone B, Epirubicin, Doxorubicin, and the combinations of Epirubicin/Taxol, Epirubicin/Epothilone A, and Epirubicin/Epothilone B, respectively. Similar treatments were performed with the non-metastatic breast cancer cell line MCF-7 (Fig. 4A), with the highly metastatic MDA-MB-231 cell line (Fig. 4B) and with normal post-selection HMEC of passage 16 (Fig. 5), respectively. Incubation with a single dose of 1 μM (blue bars) and 125 nM (red bars) of Taxol, epothilones or the anthracyclins and combinations for 6d were less effective as compared to a sequential incubation, whereby the same compounds with the same concentrations of 1 μM (yellow bars) and 125 nM (turquoise bars) were replaced after 3d, resulting in a similar 6d (= 2× 3d) incubation period, respectively. Moreover, the lower concentrated drugs (125 nM) were less effective than the 1 μM dose of these compounds, respectively. In contrast, Epothilone A and B displayed different effects in both HBCEC populations. Thus, a sequential dose of these two compounds significantly increased the cytotoxicity in one population (Fig. 3B), whereas little if any effects were observed in HBCEC from a different breast cancer patient, respectively (Fig. 3A). Similarly, Epothilone A and B exhibited different effects on the two breast carcinoma cell lines (Fig. 4A, B). Moreover, the non-metastatic MCF-7 cell line displayed an overall increased sensitivity to the administered drugs or drug combinations as compared to the highly metastatic MDA-MB-231 cells (Fig. 4A, B). Normal post-selection HMEC (P16) demonstrated reduced cytotoxic effects of the chemotherapeutics as compared to the HBCEC cultures (Fig. 5). These differences in response to certain anti-cancer drugs could be explained by the reduced or ceased proliferative capacity of senescent post-selection HMEC (P16) in contrast to the continuous proliferation of HBCEC.

## Discussion

Protease digestion-free *ex vivo *culture of human breast cancer epithelial cells (HBCEC) from breast cancer tissue revealed a cell morphology which resembled normal human mammary epithelial cells (HMEC). A successful primary culture of individualized HBCEC requires the immediate placement of a sterile biopsy from the tumor tissue in the appropriate culture medium to avoid further lesions and cell damage by the air oxygen. HBCEC were growing *in vitro *within a three-dimensional cellular network with numerous desmosomal contacts, which may be supported by desmosomal cadherins [17]. The appearance of other populations, e.g. fibroblasts or myoepithelial cells remained undetectable and further characterization of HBCEC revealed a predominant co-expression of cytokeratins and vimentin within the tumor-derived cells. Indeed, previous work has documented that culture of epithelial cells derived from solid tumors can express both, cytokeratin and vimentin intermediate filaments [1,19], whereas vimentin expression *in vivo *could differ from the *in vitro *culture [20,21].

The expression of certain cell surface marker proteins, CD24, CD44 and CD227, was maintained during long term tissue culture-derived HBCEC, demonstrating that the extended culture conditions of the tumor tissue did not affect the expression of these adhesion molecules in the HBCEC. Several studies demonstrated an association of the hetreodimeric CD227 (MUC1) with breast cancer development, whereby MUC1 is involved in the regulation of the p53 gene and is aberrantly glycosylated in mammary tumors [22-24]. Moreover, this transmembrane protein served to identify certain luminal epithelial progenitor cells in the mammary tissue [25]. In addition, mammary epithelial cells could be separated from non-epithelial cells by CD24 expression and populations expressing CD24^high ^were more precisely distinguished as luminal epithelial cells [26]. This mucin-like adhesion molecule was also shown to be associated with tumor progression and metastasis, as it was identified as a ligand of the endothelial P-selectin [27,28], and was discussed as a marker of malignancy and poor prognosis [28]. CD44 represents a proteoglycan-rich surface protein that is involved in numerous signaling mechanisms and contributes to processes such as cell adhesion, migration and invasion [29] and thus, the characterization of a distinct population of highly tumorigenic breast cancer cells revealed CD44 expression [30,31]. Of interest, certain expression levels of CD24 and CD44 are considered as breast cancer stem cell markers [32] and a significant reduction of CD24 and CD44 surface markers is observed during HMEC aging [33]. Together, the expression of CD44, CD24 and CD227 indicated a malignant potential of HBCEC which is also supported by the detection of telomerase activity. Whereas the lack of telomerase activity in normal somatic cells induces chromosomal instability followed by cell cycle arrest and cellular senescence [34], cancer cells regain activity of telomerase reverse transcriptase (hTERT) and overcome this proliferation barrier [35]. In this context, staining for the aging marker SA-β-gal after 722d of tumor tissue culture revealed hardly any senescent cells in the HBCEC population in contrast to normal senescent post-selection HMEC in passage 16, which exclusively exhibited enlarged positive cells already after 32d in culture.

Chemosensitivity assays verified an enhanced responsiveness of HBCEC to different chemotherapeutic compounds as compared to the growth-arrested normal HMEC P16. These effects revealed a specific sensitivity to the microtubule-targeting agents Epothilone A and Epothilone B, which are used predominantly for the treatment of metastatic breast cancers [36]. Taxanes also stabilize the microtubule assembly and can thereby inhibit mitosis of the tumor cells, however, resistance to taxanes can be overcome by epothilone treatment, evolving a different antitumor mechanism [37,38]. The variable reactions of distinct HBCEC populations to Epothilone A and partially Epothilone B indicated certain tumor-specific responsiveness in individual patients.

## Conclusion

Taken together, the morphological evaluation and cytokeratin expression revealed epithelial-like cells in the primary tumor tissue-derived cultures without a significant contamination of other cell types. Moreover, long term culture of the tumor biopsies revealed HBCEC populations expressing certain precursor cell-like and tumor-associated markers, including CD24, CD44 and CD227, respectively, which was paralleled by little if any senescence and a detectable telomerase activity. Finally, the HBCEC responded to chemotherapeutic agents used for breast cancer treatment, although a distinct responsiveness could be observed among individual HBCEC populations. Collectively, these findings suggest, that the successful long term culture of tumor tissue to obtain primary HBCEC contributes to optimize an individualized therapeutic approach. Thus, a representative number of these individual HBCEC cultures could provide a suitable screening platform for potentially new breast cancer therapeutics. Moreover, the long term culture of tumor tissue to obtain primary HBCEC also exhibits the opportunity to investigate metabolic and functional alterations of the tumor, including the characterization of putative biomarkers, understanding the mechanism of tumor progression and consequently, to examine the potential for developing metastatic capacity, e.g. lymph node metastases.

## Abbreviations

(ECM): extracellular matrix; (HBCEC): human breast cancer-derived epithelial cells; (HMEC): human mammary epithelial cells; (panCK): pan cytokeratin; (TRAP): telomeric repeat amplification protocol.

## Competing interests

The authors declare that they have no competing interests.

## Authors' contributions

RH conceived and designed the study, generated the primary cells from the tumor tissues, carried out the immune fluorescence analysis, aging studies and the chemotherapeutic assay and wrote the manuscript. CB carried out the cell surface marker analysis and contributed to the chemotherapeutic assay and statistical analysis. The authors read and approved the final manuscript.
